# Tangential volumetric modulated arc therapy technique for left-sided breast cancer radiotherapy

**DOI:** 10.1186/s13014-015-0392-x

**Published:** 2015-04-08

**Authors:** Tuomas Virén, Janne Heikkilä, Kimmo Myllyoja, Kristiina Koskela, Tapani Lahtinen, Jan Seppälä

**Affiliations:** Cancer Center, Kuopio University Hospital, PO Box 100, FI-70029 Kuopio, Finland

**Keywords:** Radiotherapy, Breast cancer, VMAT, IMRT, Low dose volume

## Abstract

**Background:**

The aim of the present study was to introduce a new restricted tangential volumetric modulated arc therapy (tVMAT) technique for whole breast irradiation and compare its dosimetric properties to other currently used breast cancer radiotherapy techniques.

**Method:**

Ten consecutive women with left-sided breast cancer were enrolled in this retrospective study. Four treatment plans were generated for each patient: 1) standard tangential field-in-field (FinF), 2) tangential intensity modulated radiotherapy (tIMRT), 3) tangential VMAT (tVMAT) with two dual arcs of 50-60° and 4) continuous VMAT (cVMAT) with a dual arc of 240°. The plans were created with Monaco® (tIMRT, tVMAT and cVMAT) and Oncentra® (FinF) treatment planning systems.

**Results:**

With both VMAT techniques significantly higher cardiac avoidance, dose coverage and dose homogenity were achieved when compared with FinF or tIMRT techniques (*p* < 0.01). VMAT techniques also decreased the high dose areas (above 20 Gy) of ipsilateral lung. There were no significant differences in the mean dose of contralateral breast between the tVMAT, tIMRT and FinF techniques. The dose coverage (V47.5 Gy) was greatest with cVMAT. However, with cVMAT the increase of contralateral breast dose was significant.

**Conclusions:**

The present results support the hypothesis that the introduced tVMAT technique is feasible for treatment of left-sided breast cancer. With tVMAT dose to heart and ipsilateral lung can be reduced and the dose homogeneity can be improved without increasing the dose to contralateral breast or lung.

## Background

Adjuvant radiotherapy (RT) after breast-conserving surgery of breast cancer decreases the rate of recurrence and increases the overall survival [1]. Unfortunately, a number of patients develop radiation related complications such as breast fibrosis, changes in the breast appearance and late pulmonary and cardiovascular complications [2-4]. Although the frequency of radiation-induced complications has decreased along the development of RT techniques, novel methods are needed for effective reduction of dose to heart and ipsilateral lung.

Dose inhomogeneity in treated breast has been reported to be the most important predictor of RT-induced toxicities such as fibrosis, erythema, moist desquamation and oedema [5]. With conventional tangential field-in-field (FinF) or wedge field techniques the dose inhomogeneities are usually unavoidable, especially in large-breasted patients. Furthermore, the increased use of hypofractionation has also increased the requirement for the dose homogeneity in breast cancer RT [6]. Simultaneously, to avoid radiation induced pulmonary complications dose volume constraints for ipsilateral lung must also be employed [5,7]. In addition to pulmonary complications, more and more concern has been addressed on cardiac mortality related to left-sided whole breast irradiation (WBI) [1,4]. Furthermore, sparing of left anterior descending coronary artery (LAD) has been recommended to avoid late coronary complications [8]. Since recommendations on safe dose levels to the heart are not available, techniques that minimize the cardiac exposure are needed [9].

Static tangential field or intensity modulated radiotherapy (IMRT) techniques are generally used for WBI. Tangential field techniques usually consist of two opposing fields and the dose intensity modulation is limited from these directions. As a consequence the avoidance of heart and ipsilateral lung might lead to compromising the target volume coverage and also result in higher dose inhomogeneities. IMRT has been shown to improve target dose coverage and minimize the dose in organs at risk [10,11]. However, IMRT techniques with multiple treatment fields have been reported to increase the low dose volumes in contra- and ipsilateral lung and contralateral breast [12,13]. Consequently, a fear of increasing the risk for second cancer has hindered the use of IMRT techniques in WBI.

Volumetric modulated arc therapy (VMAT) is a relatively new technique based on simultaneous optimization of multi leaf collimator (MLC), gantry rotation and dose rate. So far the VMAT techniques have been applied for WBI with varying results [14-16]. When compared to IMRT greater target volume coverage and homogeneity have been achieved with VMAT. In addition, VMAT has reduced ipsilateral lung doses when compared to conventional tangential field techniques [15]. However, the earlier VMAT techniques have been reported to increase the mean dose of contralateral lung, contralateral breast and heart as compared to the tangential techniques. Thus it has been concluded that the studied VMAT techniques should not be used for WBI [14]. In the previous dosimetric studies full or continuous partial arcs have been used in VMAT treatment planning [14-16]. Analogous to multiple-field IMRT plans, the use of full arcs consequently increases the irradiated volume. The aim of the present study was to introduce and dosimetrically evaluate a restricted tangential VMAT technique for left-sided breast cancer. We hypothesize that with the proposed tangential VMAT (tVMAT) technique the dose homogeneity within target volume can be increased. Furthermore, we hypothesize that radiation dose to heart and lung can be decreased without increasing low dose volume, especially in contralateral breast, when compared to conventional tangential field techniques.

## Method

### Patients

Ten patients (age 65 ± 6 years) with left-sided breast cancer without nodal involvement were selected in this retrospective study. The study was approved by the Research Ethics Committee of Northern Savo Hospital District. The patients were imaged with a CT scanner (Toshiba Aquilion LB, Toshiba Medical Systems Co., Tochigi, Japan) in treatment position (supine, arms up). CT images were acquired from the level of mandible to the basis of lungs with a slice thickness of 2 mm.

### Target and OAR delineation

The delineation of the clinical target volume (CTV) was based on the RTOG guidelines (http://www.rtog.org/corelab/contouringatlases.aspx). A margin of 5 mm was added to the CTV resulting in the planning target volume (PTV). The volumes outside the body contour and inside the lung were excluded from the PTV. Furthermore, for the plan evaluation and normalization purposes, a margin of 5 mm from skin surface was excluded from the PTV (resulting PTV_in_). PTV_in_ was also used as a target volume in the optimization of VMAT and IMRT plans to avoid overdosing the skin. Organs at risks (OAR) defined in this study were contra- and ipsilateral lung, heart, LAD, contralateral breast and normal tissue which was defined by excluding the PTV from the body surface (Body-PTV).

### Dose objectives and constraints

The prescription dose was 50 Gy in 25 fractions. The primary aim in treatment planning was to achieve 98% dose coverage of 47.5 Gy (V47.5) in the PTV. VMAT and IMRT plans were optimized in Monaco® TPS in a constrained mode and biological cost functions were used as constraints. The volumes of doses 30 Gy, 25 Gy and 2 Gy (V30, V20, V2) were minimized for heart and LAD. For ipsilateral lung volumes of doses 20 Gy, 10 Gy and 5 Gy (V20, V10 and V5) were minimized. For contralateral lung and breast the doses were optimized to be less than 5 and 2 Gy, respectively. The objective functions were specified individually for each patient to accomplish the best achievable treatment plan. For FinF plans the treatment planning goal was to achieve the same dose objectives used in the IMRT and VMAT planning.

### Treatment planning

**Figure 1:**
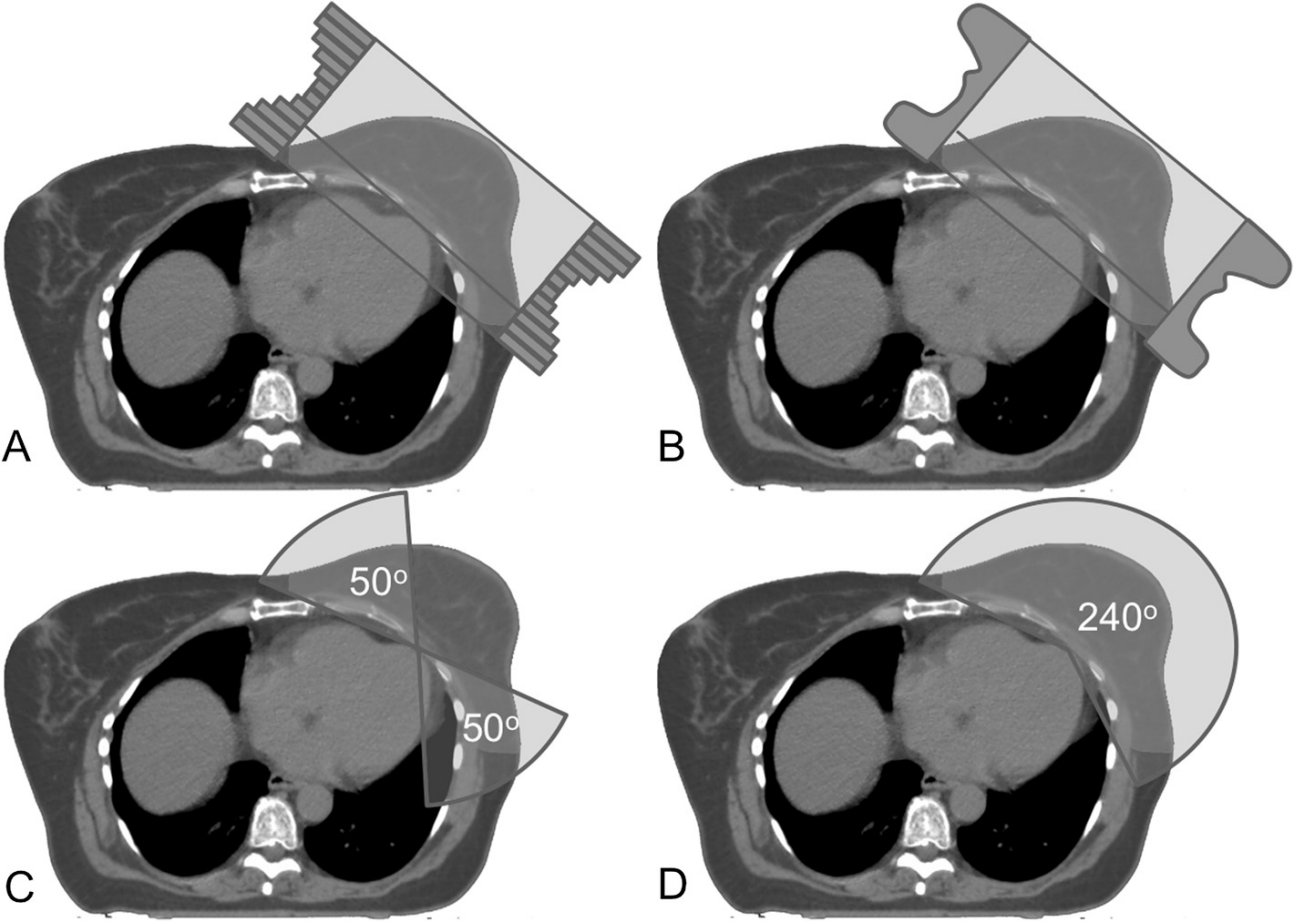
**Typical beam arrangements for FinF (A), tIMRT (B), tVMAT (C) and cVMAT (D).**

Treatment plans were created for Elekta Infinity® accelerator (Elekta AB, Stockholm, Sweden) with 5 mm Agility® MLC. The treatment energies (either 6 or 10 MV) were selected according to the size of the PTV. Four separate treatment plans with an identical isocenter were created to each patient as shown in Figure 1: 1) a standard tangential FinF, 2) a dynamic IMRT with two static tangential fields (tIMRT), 3) tangential VMAT (tVMAT) with two tangential dual arcs of 50-60° and 4) continuous VMAT (cVMAT) treatment plan with one dual arc of 240°. The tVMAT, cVMAT and tIMRT plans were generated using Monaco® treatment planning system (TPS) (version 3.30.01, Elekta AB). To determine optimal arc configuration for tVMAT and cVMAT plans several plans with different plan parameter (e.g. start and stop angles of the arcs) were generated and beam configuration that produced the best result were selected to the study. Surface margin (*e.g.* the inner margin from skin surface that is excluded from the optimization) was set to 0.5 cm. Minimum segment width of 1.0 cm and high fluence smoothing was used in the optimization. To avoid under dosing the skin optimized radiation fluences were extended 2 cm outside the body surface using auto flash operation available in Monaco® TPS. Maximum number of control points per plan was set to 170. A standard deviation of 0.5% was used in Monte Carlo (MC) dose calculation with a dose grid of 3.0 mm. The 3D-CRT FinF plans were generated in Oncentra® TPS (version 4.3.0.410, Elekta AB) with collapsed cone convolution (CCC) dose calculation algorithm with a dose grid of 3.0 mm. Doses to the OARs were minimized without compromising the PTV coverage as recommended by QUANTEC [17]. All treatment plans were normalized to the mean dose of PTV_in_.

### Plan analysis

Dose volume histograms (DVH) of the treatment plans were used in analyzing target coverage and doses to the OAR’s. In addition, the plan qualities were compared by the homogeneity index (HI) and conformity index (CI). The plan CI, defined by Paddick [18], is given by

$$ \mathrm{C}\mathrm{I}=\frac{{{\mathrm{TV}}_{\mathrm{PIV}}}^2}{\left(\mathrm{T}\mathrm{V}\cdot \mathrm{P}\mathrm{I}\mathrm{V}\right)}, $$

where the TV_PIV_ is the volume of the PTV_in_ covered by the prescription isodose, TV is the volume of the PTV_in_ and PIV the total volume covered by the prescription isodose. The HI was defined as

$$ \mathrm{H}\mathrm{I}=\frac{\left({\mathrm{D}}_1-{\mathrm{D}}_{98}\right)}{D_P}, $$

where D_1_ is the minimum dose in 1% of the PTV_in_, D_98_ is the minimum dose in 98% of the PTV_in_ and D_P_ is the prescription dose.

### Statistical analysis

One way analysis of variance with least significant difference post-hoc test (LSD) was conducted using SPSS software (version 19.0.0.2, IBM, USA) to evaluate the significance of difference between the dosimetric parameters calculated from the treatment plans. A *p*-value <0.05 was considered significant.

## Results

The VMAT and the IMRT techniques achieved significantly higher dose homogeneity (lower HI-value, *p* < 0.01) when compared with the FinF technique (Table 1). Furthermore, with the both VMAT techniques the dose homogeneity was significantly higher as compared to tIMRT (*p* < 0.05). No significant difference in CI was detected between the VMAT, IMRT or FinF techniques. The dose coverage (V47.5 Gy) was significantly superior with VMAT techniques as compared with FinF and tIMRT techniques (*p* < 0.05). There was no statistical difference in V47.5, HI or CI between tVMAT and cVMAT techniques.

**Table 1:** **Mean values and standard deviations (n = 10) of the dosimetric parameters for the different techniques studied**

	**tVMAT**	**cVMAT**	**tIMRT**	**FinF**
**PTV**				
D1cm^3^ (Gy)	53.4 ± 0.5	53.3 ± 0.4	53.7 ± 0.5	53.5 ± 0.6
V47.5 (%)	97.8 ± 0.8	98.1 ± 1.0	96.3 ± 2.0	91.4 ± 2.4
HI	0.10 ± 0.01	0.10 ± 0.01	0.12 ± 0.02	0.14 ± 0.01
CI	0.50 ± 0.03	0.50 ± 0.04	0.45 ± 0.05	0.47 ± 0.09
**Lung, Ipsilateral**				
V20 (%)	18.1 ± 5.3	15.3 ± 4.6	21.6 ± 4.0	21.4 ± 3.7
V10 (%)	25.3 ± 5.6	23.4 ± 5.6	26.2 ± 4.3	25.7 ± 3.8
V5 (%)	36.9 ± 5.4	35.5 ± 5.6	33.3 ± 4.3	32.0 ± 3.6
Mean dose (Gy)	9.6 ± 2.1	8.7 ± 1.7	10.9 ± 1.8	10.4 ± 1.5
**Lung, Contralateral**				
Mean dose (Gy)	0.9 ± 0.1	1.6 ± 0.7	0.7 ± 0.1	0.7 ± 0.1
**Heart**				
V30 (%)	5.7 ± 6.0	3.4 ± 3.9	13.9 ± 6.8	15.2 ± 6.9
V25 (%)	8.2 ± 6.9	5.0 ± 5.3	15.4 ± 7.1	16.4 ± 7.4
V2 (%)	60.8 ± 18.8	68.4 ± 22.3	61.3 ± 14.4	57.9 ± 14.4
Mean dose (Gy)	6.3 ± 3.0	5.5 ± 2.9	9.1 ± 3.4	9.1 ± 3.5
**LAD**				
V30 (%)	43.1 ± 29.5	32.0 ± 27.3	67.8 ± 19.9	79.7 ± 8.1
V25 (%)	55.0 ± 27.8	40.3 ± 28.4	72.2 ± 15.1	81.2 ± 7.5
Mean dose (Gy)	24.3 ± 8.0	20.4 ± 8.7	34.4 ± 7.0	37.9 ± 3.6
**Breast, Contralateral**				
V2 (%)	10.2 ± 7.0	53.0 ± 27.1	11.1 ± 5.6	7.0 ± 4.8
Mean dose (Gy)	1.2 ± 0.3	2.6 ± 1.2	1.0 ± 0.4	1.0 ± 0.3
**Normal tissue (Body-PTV)**				
V53.5 (cm^3^)	0.9 ± 1.1	0.4 ± 0. 5	8.5 ± 15.6	1.9 ± 4.1
V2 (%)	20.3 ± 4.6	30.4 ± 8.3	17.3 ± 2.8	15.9 ± 3.4
Mean dose (Gy)	3.3 ± 0.5	3.8 ± 0.6	3.3 ± 0.5	3.4 ± 1.1
**Body**				
Integral Dose (Gydm^3^)	105.3 ± 43.4	113.5 ± 43.8	105.6 ± 43.8	99.3 ± 46.5

**Figure 2:**
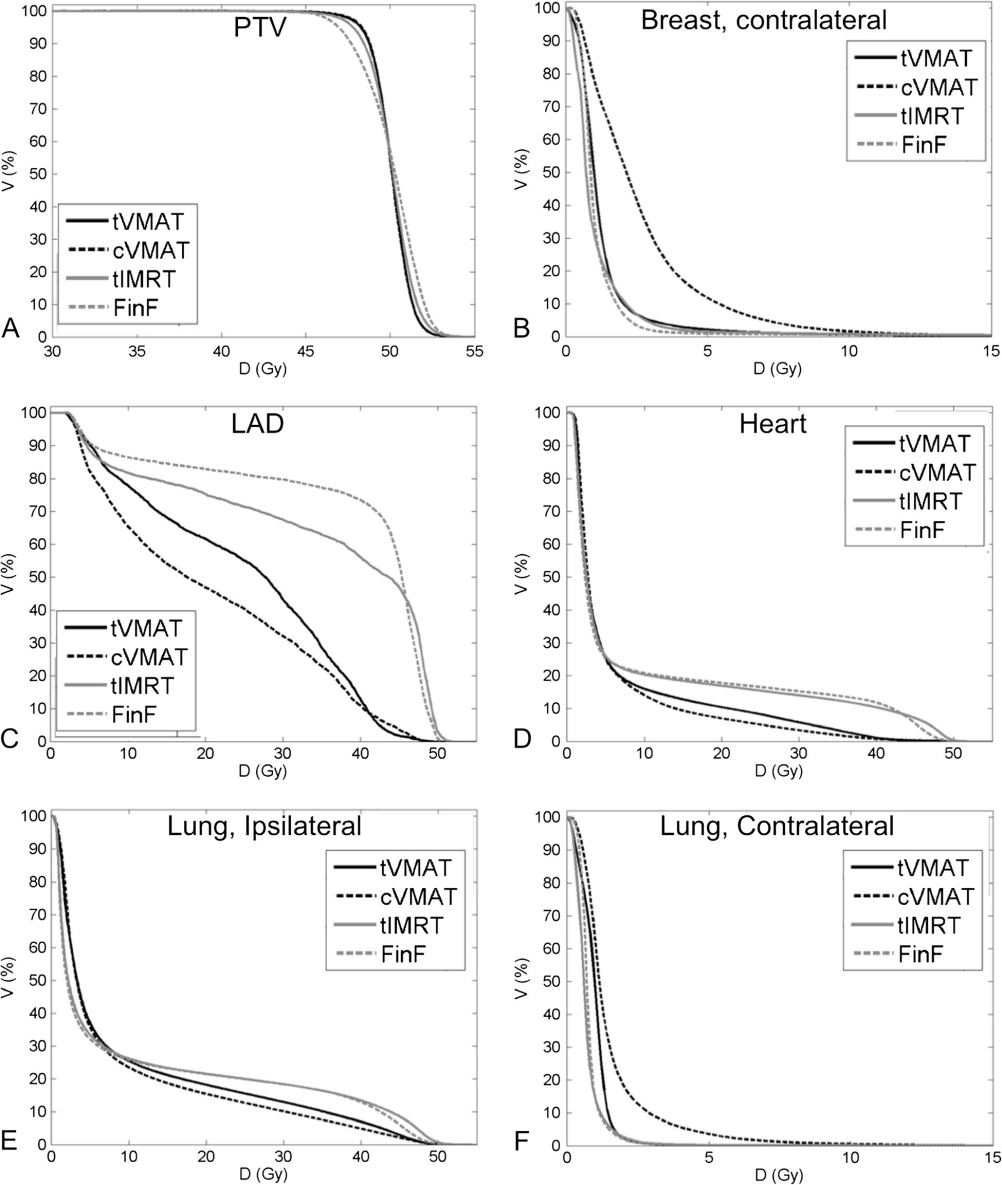
**Average cumulative DVHs (n = 10) for the four different techniques studied.** PTV_in_ **(A)**, contralateral breast **(B)**, left anterior descending coronary artery **(C)**, heart **(D)**, ipsilateral lung **(E)**, and contralateral lung **(F)**.

**Figure 3:**
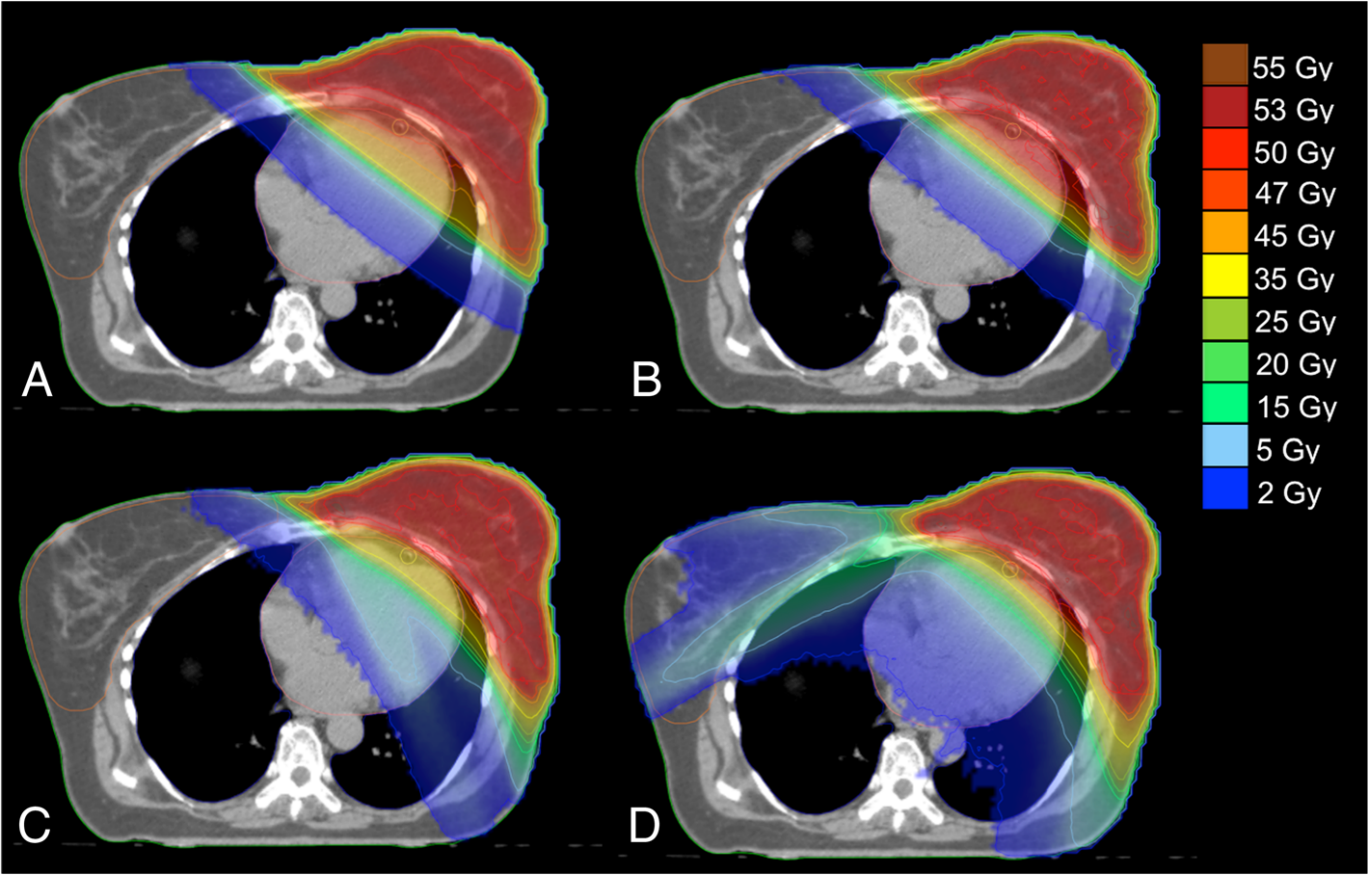
**Typical dose distributions for one patient planed with (A) FinF, (B) tIMRT, (C) tVMAT and (D) cVMAT techniques.**

Significant cardiac (heart and LAD) dose sparing was achieved with VMAT techniques when compared to FinF or tIMRT (Table 1, Figure 2, Figure 3). With tVMAT and cVMAT the V30 and V25 volumes to the heart and LAD were significantly lower when compared to IMRT or FinF techniques (*p* < 0.05) (Table 1). Furthermore, the cVMAT technique reduced the mean dose of the heart significantly when compared with tIMRT or FinF (*p* < 0.05). Slight but non-significant reduction of V2 of the heart was achieved with FinF as compared with that of tVMAT, cVMAT or tIMRT.

Significant decrease in high dose volume (V20) and mean dose of ipsilateral lung was achieved with cVMAT technique as compared with tIMRT or FinF plans (*p* < 0.01) (Table 1). Additionally, with tVMAT technique the reduction of high dose volume to the lung was evident as compared with tIMRT and FinF (Figure 2, Figure 3). However, no statistically significant differences were found in V20, V10 or V5 volumes between tVMAT, tIMRT or FinF techniques. cVMAT increased the mean dose of the contralateral lung significantly when compared with other techniques (*p* < 0.01). The difference was non-significant between tVMAT, tIMRT and FinF.

The difference in contralateral breast mean dose between tVMAT, tIMRT and FinF techniques was non-significant (Table 1, Figure 3). However, the cVMAT technique increased the mean dose of contralateral breast significantly (*p* < 0.01). No significant differences were detected in the low dose volumes (V2) to normal structures (Body-PTV) between tVMAT, tIMRT or FinF techniques as with cVMAT the increase was significant (*p* < 0.01, Table 1). No significant differences in mean dose to the normal structures or integral doses to body were detected between the techniques (Table 1). The average number of monitor units (MUs) with one fraction were on average 629 ± 93 MU, 678 ± 98 MU, 283 ± 37 MU and 233 ± 5 MU with tVMAT, cVMAT, tIMRT and FinF, respectively.

## Discussion

In this dosimetric study two different VMAT techniques were investigated for left-sided breast cancer radiotherapy and were found to increase the dose homogeneity when compared with tIMRT and FinF. Similarly, significant increases in dose homogeneity and coverage have been reported as IMRT or VMAT techniques have been compared with conventional techniques [13,14,19]. Correspondingly with the previous studies high doses to heart and LAD was avoided with the studied VMAT techniques [14,20]. As the mean dose and doses of 25 and 30 Gy to heart have been reported to be detrimental [4,17,21], it would support the potential of VMAT techniques in WBI.

In this study the high dose volumes in ipsilateral lung were decreased by using the VMAT techniques. Similar results have also been reported in previous studies in which VMAT or IMRT techniques have been compared with conventional tangential techniques [11,15,16]. However, when using the VMAT or IMRT techniques without restricting the field directions (as with cVMAT) significant increase in low dose volume has also been observed. Fear of increasing the low dose volume to lungs and contralateral breast is probably the main reason for the delayed clinical implementation of IMRT and VMAT techniques in WBI. As expected, increase in low dose volume (V5 and V2) of lungs and contralateral breast was detected with cVMAT plans when compared to tVMAT, tIMRT or FinF. Since the IMRT fields in this study were limited to opposing tangential directions no significant differences were found in contralateral lung or breast doses between tIMRT and FinF techniques. Importantly, no significant differences in mean doses to contralateral lung, and breast were detected between tVMAT and conventional tangential techniques. Furthermore, no significant differences were detected in integral dose or mean dose to normal tissue between the techniques. This indicates that there is no apparent elevation in the second cancer risk related to tVMAT technique.

Generally, mean doses to the heart, contralateral lung and breast reported in the present study are higher than those reported in some of the previous studies [11,14]. The main reason for this is the target delineation protocol used. In the current study the CTVs were delineated strictly based on the RTOG guidelines resulting in significantly larger target volumes (mainly to the medial direction) than we would have used clinically. However, an important conclusion from this study is that both the studied VMAT techniques decreased the heart doses when compared to conventional tangential techniques.

One of the limitations of this study is that the possible movement of the breast during the treatment was not accounted for. However, in recent dosimetric study no significant differences were detected in the dose homogeneity between IMRT and tangential plans using a moving target [22]. In addition, the VMAT treatment plans in this study were optimized with low beam modulation avoiding sharp dose gradients inside the target volume thus minimizing the effect of breathing motion to the dose distributions. It should be also noted that as two different dose calculation algorithms (CCC and MC) where used in the present study, part of the differences in the low dose areas might result from the accuracy of the used algorithms [23]. However, the dose distributions between the VMAT and IMRT treatment plans are directly comparable, since they were planned and calculated with the same TPS with the same dose calculation algorithm.

In the present study patients were treated in supine position. To reduce the dose to ipsilateral lung and heart, and to increase the dose homogeneity in PTV prone treatment position has been proposed for patients with large or pendulous breasts [24]. In the recent studies, in which prone treatment position was compared with supine position, significant decrease in ipsilateral lung dose was reported [25,26]. However, no significant differences were detected in doses to heart between the treatment positions [25,26]. As the heart typically moves towards chest wall in prone position usage of VMAT techniques to decrease the dose to heart might be beneficial also in prone position. However, further studies are needed to evaluate the potential of VMAT techniques in prone treatment position.

To further avoid the irradiation of heart deep-inspiration breath-hold techniques have been implemented and introduced for WBI [27]. The use of VMAT techniques could even further decrease the doses to cardiac tissue and ipsilateral lung in deep-inspiration breath-hold treatments. Unfortunately, there are a number of patients that cannot hold their breath for long times or are having difficulties to manage the breath-hold techniques. For those patients the VMAT techniques should definitely be considered to avoid irradiating high doses to heart. However, it should be noted that even if large heart doses are avoided by using breath-hold techniques significant increase in the dose coverage and homogeneity can be achieved with VMAT. The increase in dose homogeneity not only decreases the possibility of developing adverse effects in breast tissue but also allows the use of hypofractionation even for the patients with a large PTV. It should also be noted that with the restricted tVMAT technique the low dose volume is not increased in contralateral breast.

## Conclusion

The restricted tVMAT technique is an effective method for achieving a homogeneous dose coverage simultaneously reducing doses to heart, LAD and ipsilateral lung. Although, the cVMAT technique achieved the highest reduction of high dose volume in heart, LAD and ipsilateral lung, it also increased the low dose volumes of lung and contralateral breast. Since the tVMAT technique does not increase the low dose volumes in the contralateral breast or lung and significantly increases the dose coverage and homogeneity, the tVMAT technique could be a potential option for conventional WBI techniques. However, further studies evaluating the clinical outcome of treatments given with tVMAT technique is needed to proof the clinical value of the technique. All patients included in the present study were treated with tVMAT technique.

## References

[1] Clarke M, Collins R, Darby S, Davies C, Elphinstone P, Evans E (2005). Effects of radiotherapy and of differences in the extent of surgery for early breast cancer on local recurrence and 15-year survival: an overview of the randomised trials. Lancet.

[2] Mukesh MB, Harris E, Collette S, Coles CE, Bartelink H, Wilkinson J (2013). Normal tissue complication probability (NTCP) parameters for breast fibrosis: Pooled results from two randomised trials. Radiother Oncol.

[3] Blom Goldman U, Wennberg B, Svane G, Bylund H, Lind P (2010). Reduction of radiation pneumonitis by V20-constraints in breast cancer. Radiat Oncol.

[4] Darby SC, Ewertz M, McGale P, Bennet AM, Blom-Goldman U, Brønnum D (2013). Risk of ischemic heart disease in women after radiotherapy for breast cancer. N Engl J Med.

[5] Tortorelli G, Di Murro L, Barbarino R, Cicchetti S, Di Cristino D, Falco MD (2013). Standard or hypofractionated radiotherapy in the postoperative treatment of breast cancer: a retrospective analysis of acute skin toxicity and dose inhomogeneities. BMC Cancer.

[6] Yarnold J, Bentzen SM, Coles C, Haviland J (2011). Hypofractionated whole-breast radiotherapy for women with early breast cancer: myths and realities. Int J Radiat Oncol Biol Phys.

[7] Yorke ED, Jackson A, Rosenzweig KE, Braban L, Leibel SA, Ling CC (2005). Correlation of dosimetric factors and radiation pneumonitis for non-small-cell lung cancer patients in a recently completed dose escalation study. Int J Radiat Oncol Biol Phys.

[8] Nilsson G, Holmberg L, Garmo H, Duvernoy O, Sjögren I, Lagerqvist B (2012). Distribution of coronary artery stenosis after radiation for breast cancer. J Clin Oncol.

[9] Chung E, Corbett JR, Moran JM, Griffith KA, Marsh RB, Feng M (2013). Is there a dose–response relationship for heart disease with low-dose radiation therapy?. Int J Radiat Oncol Biol Phys.

[10] Hurkmans CW, Cho BCJ, Damen E, Zijp L, Mijnheer BJ (2002). Reduction of cardiac and lung complication probabilities after breast irradiation using conformal radiotherapy with or without intensity modulation. Radiother Oncol.

[11] Hong L, Hunt M, Chui C, Spirou S, Forster K, Lee H (1999). Intensity-modulated tangential beam irradiation of the intact breast. Int J Radiat Oncol Biol Phys.

[12] Tsuchiya K, Kinoshita R, Shimizu S, Nishioka K, Harada K, Nishikawa N (2014). Dosimetric comparison between intensity-modulated radiotherapy and standard wedged tangential technique for whole-breast radiotherapy in Asian women with relatively small breast volumes. Radiol Phys Technol.

[13] Dogan N, Cuttino L, Lloyd R, Bump E, Arthur DW (2007). Optimized dose coverage of regional lymph nodes in breast cancer: the role of intensity-modulated radiotherapy. Int J Radiat Oncol Biol Phys.

[14] Jin G-H, Chen L-X, Deng X-W, Liu XW, Huang Y, Huang XB (2013). A comparative dosimetric study for treating left-sided breast cancer for small breast size using five different radiotherapy techniques: conventional tangential field, filed-in-filed, Tangential-IMRT, Multi-beam IMRT and VMAT. Radiat Oncol.

[15] Johansen S, Cozzi L, Olsen DR (2009). A planning comparison of dose patterns in organs at risk and predicted risk for radiation induced malignancy in the contralateral breast following radiation therapy of primary breast using conventional, IMRT and volumetric modulated arc treatment technique. Acta Oncol.

[16] Popescu CC, Olivotto IA, Beckham WA, Ansbacher W, Zavgorodni S, Shaffer R (2010). Volumetric modulated arc therapy improves dosimetry and reduces treatment time compared to conventional intensity-modulated radiotherapy for locoregional radiotherapy of left-sided breast cancer and internal mammary nodes. Int J Radiat Oncol Biol Phys.

[17] Gagliardi G, Constine LS, Moiseenko V, Correa C, Pierce LJ, Allen AM (2010). Radiation dose-volume effects in the heart. Int J Radiat Oncol Biol Phys.

[18] Paddick IA (2000). Simple scoring ratio to index the conformity of radiosurgical treatment plans, Technical note. J Neurosurg.

[19] Qiu J-J, Chang Z, Wu QJ, Yoo S, Horton J, Yin FF (2010). Impact of Volumetric Modulated Arc Therapy Technique on Treatment With Partial Breast Irradiation. Int J Radiat Oncol.

[20] Sakumi A, Shiraishi K, Onoe T, Yamamoto K, Haga A, Yoda K (2012). Single-Arc Volumetric Modulated Arc Therapy Planning for Left Breast Cancer and Regional Nodes. J Radiat Res.

[21] Gagliardi G, Lax I, Söderström S, Gyenes G, Rutqvist LE (1998). Prediction of excess risk of long-term cardiac mortality after radiotherapy of stage I breast cancer. Radiother Oncol.

[22] Liu Q, McDermott P, Burmeister J (2007). Effect of respiratory motion on the delivery of breast radiotherapy using SMLC intensity modulation. Med Phys.

[23] Heikkilä J, Seppälä J, Virén T, Lahtinen T, Heikkilä J, Seppälä J (2014). Accuracy of contralateral breast doses using different treatment planning algorithms.

[24] Merchant TE, McCormick B (1994). Prone position breast irradiation. Int J Radiat Oncol Biol Phys.

[25] Mulliez T, Speleers B, Madani I, De Gersem W, Veldeman L, De Neve W (2013). Whole breast radiotherapy in prone and supine position: is there a place for multi-beam IMRT?. Radiat Oncol.

[26] Krengli M, Masini L, Caltavuturo T, Pisani C, Apicella G, Negri E (2013). Prone versus supine position for adjuvant breast radiotherapy: a prospective study in patients with pendulous breasts. Radiat Oncol.

[27] Mast ME, Van Kempen-Harteveld L, Heijenbrok MW, Kalidien Y, Rozema H, Jansen WP (2013). Left-sided breast cancer radiotherapy with and without breath-hold: Does IMRT reduce the cardiac dose even further?. Radiother Oncol.

